# Reverse remodeling of tricuspid valve morphology and function in chronic thromboembolic pulmonary hypertension patients following pulmonary thromboendarterectomy: a cardiac magnetic resonance imaging and invasive hemodynamic study

**DOI:** 10.1186/s12872-021-02248-3

**Published:** 2021-09-17

**Authors:** Christian Alcaraz Frederiksen, Farhad Waziri, Steffen Ringgaard, Søren Mellemkjær, Tor Skibsted Clemmensen, Vibeke Elisabeth Hjortdal, Sten Lyager Nielsen, Steen Hvitfeldt Poulsen

**Affiliations:** 1 Department of Cardiology, Aarhus University Hospital, Aarhus, Denmark; 2 Department of Clinical Medicine, Aarhus University, Aarhus, Denmark; 3 The MRI Research Centre, Aarhus University Hospital, Aarhus, Denmark; 4 Department of Cardiothoracic Surgery, Rigshospitalet, Copenhagen, Denmark

**Keywords:** Cardiac magnetic resonance imaging, Tricuspid valve, Chronic thromboembolic pulmonary hypertension

## Abstract

**Background:**

To investigate changes in tricuspid annulus (TA) and tricuspid valve (TV) morphology among chronic thromboembolic pulmonary hypertension (CTEPH) patients before and 12 months after pulmonary thromboendarterectomy (PEA) and compare these findings to normal control subjects.

**Methods:**

20 CTEPH patients and 20 controls were enrolled in the study. The patients were examined with echocardiography, right heart catherization and cardiac magnetic resonance imaging prior to PEA and 12 months after.

**Results:**

Right atrium (RA) volume was significantly reduced from baseline to 12 months after PEA (30 ± 9 vs 23 ± 5 ml/m^2^, *p* < 0.005). TA annular area in systole remained unchanged (*p* = 0.11) and was comparable to controls. The leaflet area, tenting volume and tenting height in systole were significantly increased at baseline but decreased significantly with comparable values to controls after 12 months (*p* < 0.005). There was correlation between the changes of right ventricular-pulmonary artery coupling and changes of TV tenting height (r = − 0.54, *p* = 0.02), TV tenting volume (r = − 0.73, *p* < 0.001) and TV leaflet area (− 0.57, *p* = 0.01) from baseline to 12 months after PEA. Tricuspid regurgitation jet area/RA area was significantly (*p* < 0.01) reduced from baseline (30 ± 13%) to 12 months after PEA (9 ± 10%).

**Conclusion:**

In CTEPH patients selected for PEA, TV tenting height, volume and valve area are significantly increased whereas annulus size and shape are less affected. The alterations in TV morphology are fully reversed after PEA and correlates to improvements of right ventricular-pulmonary arterial coupling.

## Background

Functional tricuspid regurgitation (TR) develops as a consequence of geometrical distortion in the anatomical structures situated in relation to the tricuspid valve (TV) causing leaflet tethering and tricuspid annular (TA) dilatation [1]. Functional TR is often secondary to left sided valve disease or chronic atrial fibrillation [2, 3]. Pulmonal arterial hypertension (PH) is another important cause of functional TR that might lead to excessive right ventricle (RV) remodeling and dysfunction. The subsequent annular and atrial dilatation with development of right sided heart failure carries a poor prognosis [4, 5]. Reduction of PH is associated with TR severity regression in contrast to progression of PH that is associated with an increase of TR severity which is associated with poor survival [6]. In patients with worsening PH progressive TR development is shown to relate to RV enlargement and increased RV sphericity leading to TA dilatation and increased TV tethering [6]. Patients with chronic thromboembolic pulmonary hypertension (CTEPH) suffer from varying degrees of functional TR in combination with right sided heart failure. Following successful pulmonary thromboendarterectomy (PEA) an immediate reduction in pulmonary artery (PA) pressures occurs [7, 8]. Early post-operative examination by two-dimensional (2D) echocardiography and right heart catherization have demonstrated that reduction in PA systolic pressures after PEA is associated with a significant 70% reduction in the number of patients with severe TR. This reduction occurs despite of persistent tricuspid annular dilation [8]. However, the long-term effects on TR severity and detailed changes in TV morphology in relation to changes of pulmonary pressures, RV-PA coupling, and RV/TA remodeling have not been studied in detail. The assessment of RV and TV function including evaluation of TR severity is routinely performed by 2D echocardiographic examination which has some limitations due to the inherent geometrical anatomic characteristics of the right side of the heart [9]. Transthoracic three-dimensional (3D) echocardiography offers accurate data on RV volumes, function and valve morphology. However, precise 3D echocardiographic imaging of the TV can be technically challenging and is difficult to obtain in all patients. Cardiac Magnetic Resonance Imaging (CMR) is considered as the gold standard for accurate assessment of RV and RA dimension, volumes and function.

In the present study, we aimed to investigate the changes of TA and TV morphology assessed by CMR with relation to changes in pulmonary arterial pressures by right heart catherization (RHC) in CTEPH patients before and 12 months after PEA and compare these findings to normal control subjects.

## Methods

### Patients

Between December 2014 and January 2017, we enrolled 20 CTEPH patients and 20 controls in the study at Aarhus University Hospital, Denmark. In total, 47 consecutive patients were evaluated for CTEPH during the study period. After clinical and diagnostic evaluation 20 patients were identified to fulfill the inclusion criteria for participation in in the study, which included: Established CTEPH diagnosis, age ≥ 18 years and indication for PEA [10, 11]. The reasons for exclusion were as follows: five patients declined to participate, five patients did not meet the diagnostic criteria of CTEPH (three idiopathic pulmonary arterial hypertension, one idiopathic pulmonary fibrosis and one with constrictive pericarditis); five patients had only borderline PH and received medical treatment, four patients were deemed inoperable or excluded due to co-existing comorbidities and eight were excluded for various other reasons.

The CTEPH diagnosis was defined in accordance with the World Health Organization classification as PH with a mean PA pressure (mPAP) ≥ 25 mmHg, a pulmonary capillary wedge pressure ≤ 15 mmHg, and specific angiographic signs at least 3 months after effective anticoagulation [12]. The CTEPH patients were examined with RHC, transthoracic echocardiography (TTE) and CMR on the same day, prior to PEA and 12 months after PEA. The controls were enrolled from all parts of Denmark through an online recruitment website (forsoegsperson.dk), to ensure that the controls represented the general population. The control group participated on a volunteer basis and prior to inclusion they underwent screening to exclude cardiovascular disease with medical history, ECG, TTE and blood pressure measurement. They were required to be asymptomatic and received no medication. All examinations including CMR was performed on the same day.

The PEA procedure was performed on cardiopulmonary bypass in deep hypothermia and periods of circulatory arrest. None of the patients underwent concomitant coronary artery bypass grafting or valve interventions.

### Cardiac magnetic resonance

CMR was performed using a Philips Achieva dStream 1.5 T whole body MR scanner (Philips Medical Systems, Best, Netherlands). Cine scanning (time-resolved imaging) was used to assess right atrium (RA) and ventricle and left atrium and ventricle volumes and geometry. An integrated 4-electrode electrocardiogram (ECG) was used to synchronize data acquisition. A survey scan was followed by an ECG-triggered temporally resolved cine scan using a balanced steady-state free precession sequence during breath-hold. The following imaging parameters were used: repetition time 2.9 ms, echo time 1.46 ms, flip angle 90°, 160 × 136 acquisition matrix, 30 phases within one cardiac cycle, slice thickness 8 mm and field of view 319 × 319 mm.

A stack of 3 long-axis slices was acquired for 4-chamber view, and for short-axis view 10–12 slices were acquired covering the entire right and left ventricle. For tricuspid valve analysis a special focused stack specifically designed for this study consisting of 6–8 long-axis slices and 10–12 slices in short-axis were acquired covering the entire tricuspid valve and right ventricle. Pulmonary flow measurements were obtained using a free-breathing, ECG-triggered phase contrast sequence. The image parameters for the phase contrast sequence were: slice thickness 7 mm, 301 × 301 mm field of view, 128 × 96 acquisition matrix, 35 cardiac frames, velocity encoding 150 cm/s and scan duration 1:43 min. Image analysis of the heart chambers and pulmonary flow were performed using Segment v.2.2 R6274 (Medviso AB, Lund, Sweden). Tricuspid valve analyses were performed in an inhouse software originally developed for detailed mitral valve analyses as described previously [13] (Siswin, Aarhus, Denmark).

**Fig. 1:**
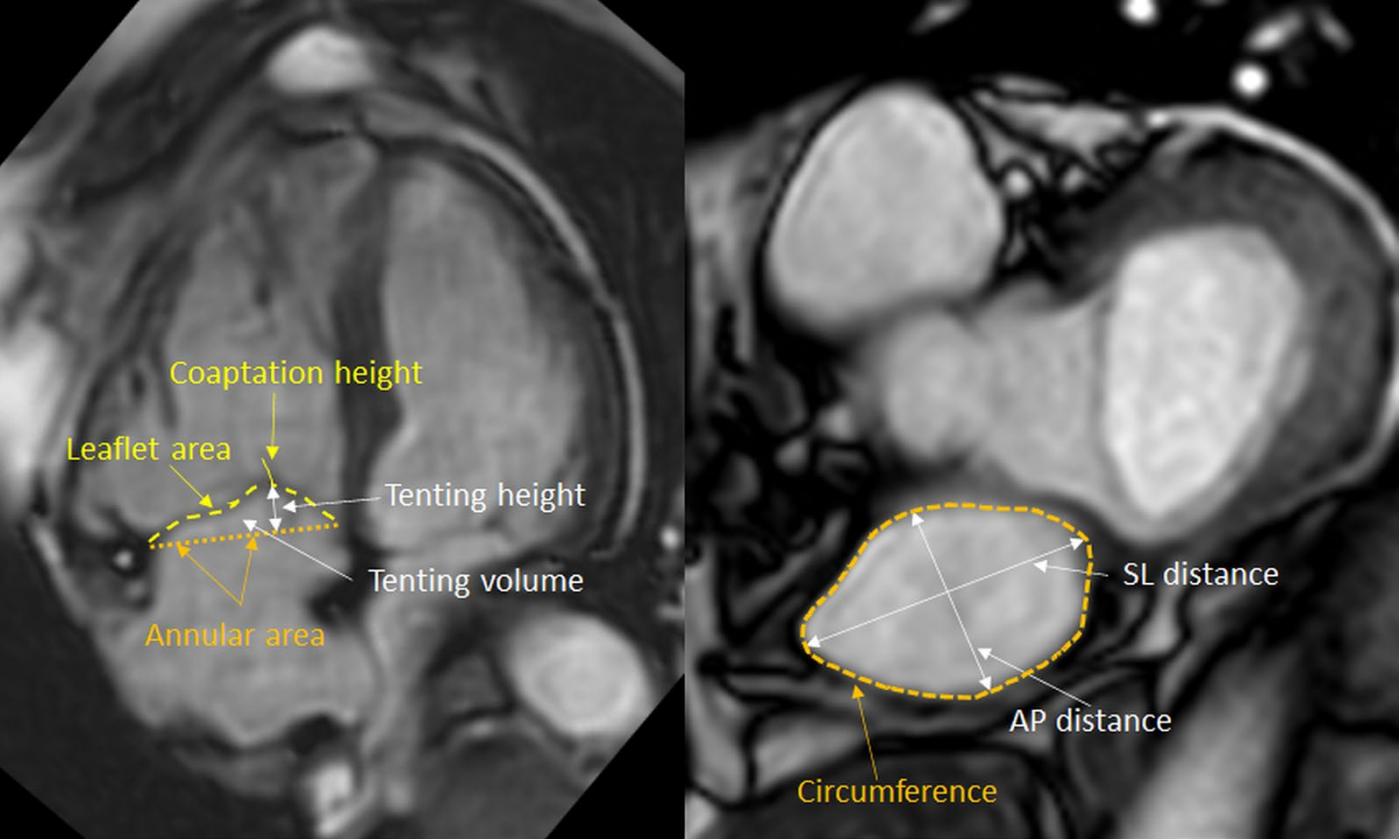
CMR images used for detailed analyses of the tricuspid valve. *Left panel*. Modified 4-chamber view illustrating the definition of coaptation height, leaflet area, tenting height, tenting volume, annular area. *Right panel*. Modified short axis view illustrating the definition of circumference, AP distance, SL distance

Anatomical details of the TV and TA were measured by manually tracing multiple slices through the stack. This allowed for the construction of a 3-dimensional model of the valve and the annulus. The annular area was defined by projecting annular points to the same fitted plane. Annular height was defined as the maximal distance between annular points in the plane defining the annular area. A schematic overview of the measured parameters is illustrated in Fig. 1.

RA area and volume were assessed from 4-chamber view by manual detection of endocardial border. RA length and width were assessed from 4-chamber view by measuring the inner atrial border distance in longitudinal and transverse direction, respectively. The sphericity index of the RV is estimated in the 4-chamber view as the ratio of the short diameter (RVDd1) and RV length in end-diastole. RV volumes were calculated from the short-axis cine images. From the stack of parallel short-axis images, end-diastolic volume (EDV), end-systolic volume (ESV), and stroke volume (SV) and ejection fraction were calculated. RV mass was measured in systole. Cardiac output (CO) was measured as pulmonary flow. CO was indexed to body surface area as cardiac index (CI).

### Transthoracic echocardiography

Transthoracic echocardiography was performed using a Vivid E95 ultrasound system (GE Healthcare, Horten, Norway) equipped with an M5S transducer.

Color Doppler imaging was used to assess severity of the TR, and the transvalvular gradient was measured using continuous‐wave Doppler. Quantification of TR severity was done in accordance with guidelines using the proximal isovelocity surface area method and estimation of TR jet area/RA area [9].

The echocardiographic data were blinded to invasive measurements and clinical status and examined by a single investigator. Data were analyzed offline using dedicated software (EchoPAC version 213, GE Healthcare, Horten, Norway).

### Right heart catheterization

Standard RHC was performed using a standard 7.5F triple-lumen Swan-Ganz catheter (Edwards Lifesciences, Irvine, CA, USA). The following parameters were measured: mean RA pressure (mRAP), systolic, diastolic and mPAP, mean pulmonary capillary wedge pressure (PCWP), CO, CI and systemic blood pressure. PCWP was measured at end expiration. CO was measured according to the Fick principle. RV contractility was also quantified by maximum/end-systolic elastance (Ees) and RV afterload with arterial elastance (Ea). Ees was calculated as (RV maximum pressure − mPAP) divided by SV [14, 15]. Ea was estimated by the ratio of mPAP to SV [15]. Since healthy controls were not examined with RHC, RV-pulmonary arterial (PA) coupling was assessed using only the volume method (Ees/Ea = RV-SV/RV-end systolic volume) [15, 16]. For comparison between the CTEPH patient group and healthy control group we used the RV volume method.

### Statistical analysis

Data are presented as mean ± standard deviation, unless otherwise specified. Histograms and Q-Q-plots were used to check for normality. Between-group differences were assessed by t-test for normally distributed data and Mann–Whitney U-test for non-normally distributed data. Baseline versus 12 months differences were assessed by the paired sample t-test and Wilcoxon signed-rank test for non-normally distributed data. Pearson's correlation coefficient was calculated for normally distributed data and Spearman correlation coefficient for non-normally distributed data. Categorical variables were compared using the chi-square test. Analyses were performed using Stata (STATA/IC 14.2, StataCorp LP, College Station, TX, USA).

## Results

Baseline characteristics of 20 CTEPH patients and 20 control subjects are shown in Table 1.

**Table 1:** Patient characteristics

	Baseline (n = 20)	Controls (n = 20)	*p* value
Age (years)	61 ± 14	54 ± 9	0.07
Female, n (%)	13 (65)	10 (50)	0.34
BMI (kg/m^2^)	28 ± 6	24 ± 4	< 0.05
NYHA I/II/III/IV, n (%)	0/3/16/1(0/15/80/5)		
Arterial hypertension, n (%)	9 (45)		
Atrial fibrillation, n (%)	3 (15)		
COPD, n (%)	7 (35)		
Diabetes mellitus, n (%)	1 (5)		
Ischemic heart disease, n (%)	0		
Stroke, n (%)	1 (5)		
Biochemistry			
NT-ProBNP (ng/l)	890 [430; 2692]		
Hemoglobin (mmol/l)	8.9 ± 1.4		
Creatinine (µmol/l)	85 ± 19		
eGFR (ml/min)	68 ± 14		
Medicine			
Warfarin, n (%)	20 (100)		
Aspirin, n (%)	2 (10)		
ACE/ATII inhibitor, n (%)	9 (45)		
Loop-diuretic, n (%)	5 (25)		
Thiazide, n (%)	4 (20)		
Spironolactone, n (%)	2 (10)		
Sildenafil, n (%)	1 (5)		
Riociguat, n (%)	1 (5)		

Data are presented as an absolute number and (percent) or mean ± standard deviation or median and [interquartile range]. BMI: Body Mass Index, COPD: Chronic obstructive pulmonary disease, NYHA: New York Heart Association

At baseline, CTEPH patients were highly symptomatic with the majority in New York Heart Association (NYHA) class III. NYHA class improved 12 months after PEA (NYHA class I-II (n = 19) and III-IV (n = 1)) (*p* < 0.0001). A significant reduction in Nt-ProBNP was also observed: preoperatively 890 [430; 2692] ng/l to 239 [141; 375] ng/l at 12 months (*p* < 0.005). Mild TR was noted in 60% and moderate-severe TR was noted in 40% of the patients at baseline with an average effective regurgitant orifice (ERO) of 0.30 ± 0.30 cm^2^ and a TR jet area/RA area of 30 ± 13% for all patients. After 12 months only two patients had moderate-severe TR with an ERO of 0.38 cm^2^ and 0.47 cm^2^ whereas 18 patients only had trace or mild TR. The TR jet area/RA area was reduced to 9 ± 10%, (vs baseline *p* < 0.01) whereas the ERO, due to very mild regurgitation, was not satisfactory obtainable in 15 patients.

### Reverse remodeling of right atrium, ventricle and tricuspid valve during follow-up by CMR

Table 2 demonstrates the variables of RA, RV and TV at baseline, at 12 months after PEA and in 20 control subjects. Considerable RV remodeling was demonstrated as RV mass index, RV volumes, dimensions and sphericity index decreased significantly and was comparable with control subjects after 12 months. RV ejection fraction was impaired significantly at baseline but despite improvement remained mildly depressed as compared to controls.

**Table 2:** Cardiac magnetic resonance imaging

	Baseline (n = 19)	12 months (n = 19)	*p* value	Controls (n = 20)
Right atrium				
Volume (mL/m^2^)	30 ± 9*	23 ± 5	< 0.005	23 ± 6
Length (mm)	57 ± 8	56 ± 10	0.54	53 ± 7
Width (mm)	57 ± 12*	46 ± 8	< 0.005	48 ± 9
Right ventricle				
RVMi (g/m^2^)	22 ± 7*	13 ± 7	< 0.0001	12 ± 3
End-diastolic volume (mL)	233 ± 74*	164 ± 50	< 0.0001	172 ± 41
End-systolic volume (mL)	168 ± 75*	94 ± 40	< 0.0005	80 ± 23
RVDd1 (mm)	54 ± 8*	42 ± 8	< 0.0001	43 ± 5
RVDd2 (mm)	48 ± 8*	38 ± 6	< 0.0001	37 ± 4
RV length (mm)	86 ± 10	78 ± 10	< 0.0001	82 ± 8
RV sphericity index	2.5 ± 0.2^*^	2.8 ± 0.5	< 0.01	2.8 ± 0.4
Stroke volume (mL)	65 ± 22*	71 ± 21*	0.15	92 ± 19
TV regurgitant volume (mL)	11 ± 17	4 ± 8	0.20	4 ± 7
Ejection fraction (%)	30 ± 13*	44 ± 10*	< 0.001	54 ± 20
Tricuspid valve				
Annular area sys (cm^2^)	12.6 ± 3.5	11.0 ± 3.2	0.11	12.4 ± 2.6
Annular area dia (cm^2^)	14.5 ± 4.2	11.7 ± 3.9*	0.06	13.5 ± 2.8
Circumference sys (cm)	15.0 ± 2.6	13.9 ± 2.8	0.18	15.0 ± 2.1
Circumference dia (cm)	16.7 ± 1.9	15.9 ± 3.0	0.47	16.7 ± 1.9
Annular height sys (cm)	1.3 ± 0.5*	1.1 ± 0.3	< 0.01	1.0 ± 0.3
Annular height dia (cm)	1.3 ± 0.4	1.7 ± 0.7	0.16	1.5 ± 0.4
SL distance sys (cm)	4.0 ± 0.6	3.8 ± 0.6	0.08	3.7 ± 0.5
SL distance dia (cm)	4.4 ± 0.6	4.2 ± 0.7	0.24	4.1 ± 0.5
AP distance sys (cm)	3.9 ± 0.5	3.8 ± 0.7	0.42	4.1 ± 0.5
AP distance dia (cm)	4.1 ± 0.6	3.7 ± 0.8*	0.08	4.2 ± 0.6
Leaflet area sys (cm^2^)	20.1 ± 5.8	16.2 ± 6.4	< 0.005	16.9 ± 3.7
Tenting volume sys (cm^3^)	4.4 ± 2.6*	1.6 ± 0.9	< 0.005	1.5 ± 0.8
Tenting height sys (mm^3^)	11.4 ± 3.9*	7.0 ± 2.2	< 0.0005	7.0 ± 1.9
Coaptation height (mm)	4.9 ± 1.1*	5.8 ± 1.7*	0.17	7.2 ± 1.5
Pulmonary artery				
Cardiac output (L/min)	3.9 ± 0.9*	5.1 ± 1.4	< 0.005	4.9 ± 0.7
Cardiac index (L/min/m^2^)	2.3 ± 0.4*	2.8 ± 0.6	< 0.005	2.6 ± 0.4
Non-invasive derived RV-PA coupling				
SV (mL) / ESV (mL)	0.49 ± 0.30*	0.84 ± 0.31*	*p* < 0.0001	1.19 ± 0.20

Data are presented as mean ± standard deviation

AP, anterior–posterior; dia, diastolic; SL, septal-lateral; sys, systolic; Ea, Arterial elastance; Ees, End-systolic elastance; RV, Right ventricular; PA, Pulmonary artery; RVD1, Basal RV linear dimension at end-diastole; RVD2, Mid-cavity RV linear dimension at end-diastole; RVMi, Right ventricular mass index; SV, Stroke volume; ESV, End-systolic volume

*p < 0.05 versus controls

The TV size by annular area and circumference did not change significantly even though a tendency towards lower annular area was noted. The SL/AP ratio in systole and diastole remained unchanged during follow-up (baseline vs 12 months: diastole: 1.07 ± 0.09 vs 1.20 ± 0.36, *p* = 0.26 and systole: 1.06 ± 0.19 vs 0.96 ± 0.15, *p* = 0.094). TA height in systole was increased at baseline but decreased significantly during follow-up and was comparable to controls. The difference between height of TA in systole and diastole changed significantly. At baseline the difference was 0.2 ± 5.1 mm compared to − 5.8 ± 7.0 mm at 12 months, *p* = 0.028. The systole-diastole difference was comparable to controls (− 4.3 ± 3.8 mm) at 12 months.

**Fig. 2:**
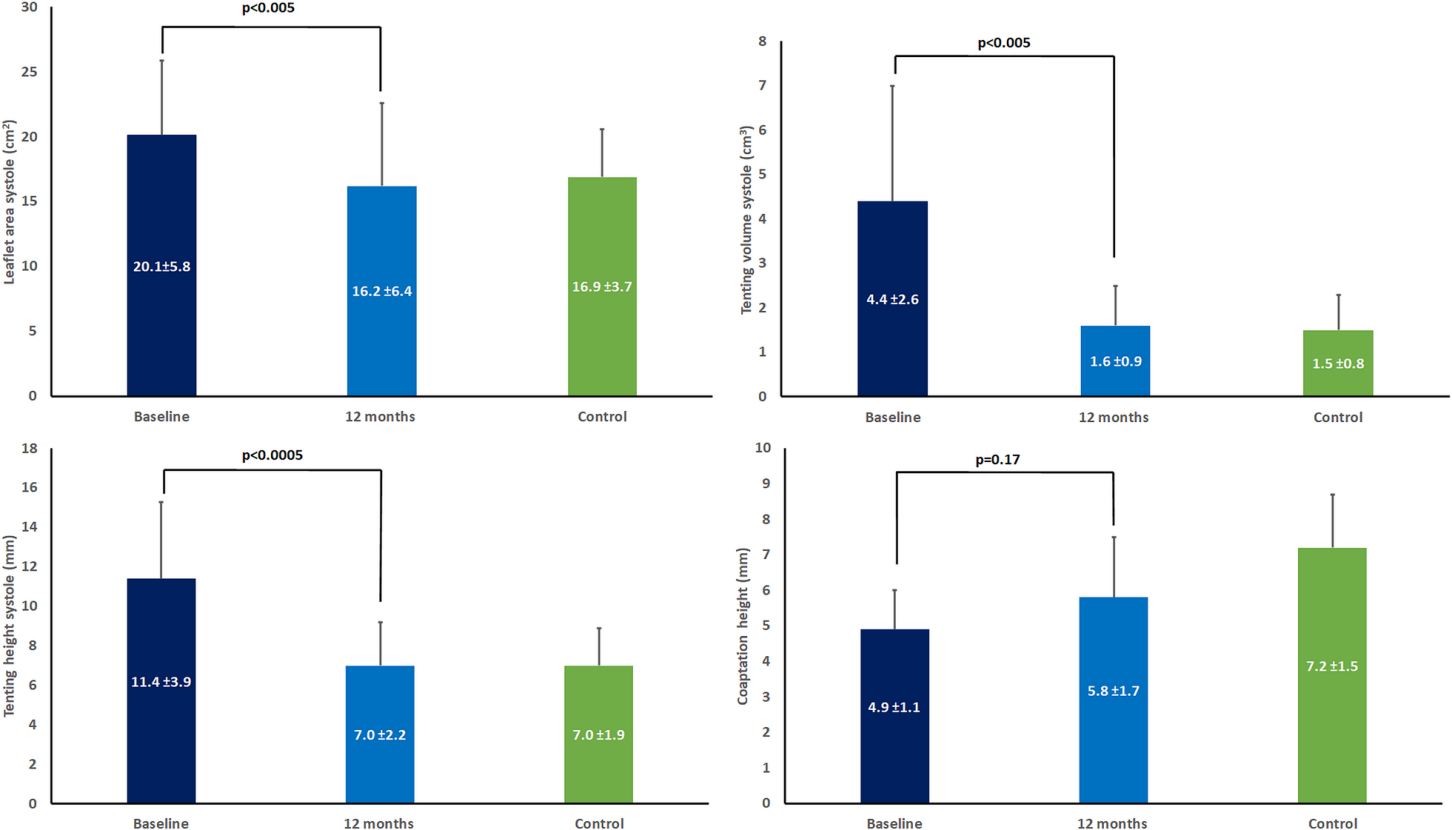
Key parameters from assessment of the tricuspid valve before and after intervention compared to controls. *Upper right panel*. Leaflet area in systole. *Lower right panel*. Tenting height in systole. *Upper left panel*. Tenting volume in systole. *Lower left panel*. Coaptation height

Changes in leaflet area, coaptation height, volume and tenting height in systole are presented in Fig. 2.

### Right heart catherization hemodynamic parameters at baseline and 12 months post-operatively

Table 3 demonstrates the hemodynamic data at baseline and after 12 months. As expected, pulmonary pressures, pulmonary vascular resistance, pulmonary end-systolic elastance, and pulmonary elastance decreased during follow-up.

**Table 3:** Invasive hemodynamic parameters

	Baseline (n = 19)	12 months (n = 19)	*p* value
Hemodynamic characteristics			
Heart rate (beats/min)	73 ± 14	76 ± 10	0.23
Systolic blood pressure (mmHg)	135 ± 18	139 ± 19	0.59
Diastolic blood pressure (mmHg)	92 ± 13	87 ± 12	0.14
mRAP (mmHg)	10.5 ± 5.1	4.3 ± 4.2	< 0.05
sPAP (mmHg)	81 ± 20	43 ± 16	< 0.0005
dPAP (mmHg)	33 ± 9	17 ± 7	< 0.0001
mPAP (mmHg)	51 ± 12	27 ± 10	< 0.0005
PCWP mean (mmHg)	11 ± 3	9 ± 4	0.21
PVR (Wood Units)	10.0 ± 4.5	3.9 ± 2.5	< 0.0005
Ees (mmHg/mL)	0.52 ± 0.29	0.23 ± 0.14	*p* < 0.0001
Ea (mmHg/mL)	0.91 ± 0.45	0.43 ± 0.25	*p* < 0.0005
Cardiac output (L/min) (Fick)	5.0 ± 2.5	5.1 ± 1.5	0.91

Data are presented as mean ± standard deviation

dPAP, Diastolic pulmonary arterial pressure; Ea, Arterial elastance; Ees, End-systolic elastance; mPAP, Mean pulmonary arterial pressure; mRAP, Mean right atrial pressure; PCWP, Pulmonary capillary wedge pressure; PVR, pulmonary vascular resistance; sPAP, Systolic pulmonary arterial pressure

### Correlations between changes of hemodynamic parameters and tricuspid valve morphology parameters

The changes of selected hemodynamic parameters and changes to TV parameters from baseline to 12 months after PEA are demonstrated in Table 4.

**Table 4:** Correlation analyzes

Hemodynamics	CMR-TV	r	*p* value
Δ SPAP	Δ Annular Area Systole	− 0.53	< 0.04
Δ SPAP	Δ SL distance	0.55	< 0.03
Δ SPAP	Δ Tenting height	0.10	0.70
Δ SPAP	Δ Coaptation height	− 0.04	0.88
Δ RA	Δ Annular area systole	− 0.60	< 0.02
Δ RA	Δ Circumference	− 0.60	< 0.02
Δ RA	Δ Tenting height	− 0.10	0.70
Δ RA	Δ Coaptation height	− 0.04	0.88
Δ CO	Δ Coaptation height	− 0.58	< 0.02
Δ Ees	Δ Annular area systole	− 0.72	< 0.003
Δ Ees	Δ SL distance	− 0.55	< 0.03
Δ Ees	Δ Annular area systole	0.26	0.29
Δ Ees	ΔSL distance	0.24	0.33
ΔRV-PA coupling	Δ Annular area systole	− 0.50	0.04
ΔRV-PA coupling	Δ SL distance	− 0.53	0.02
ΔRV-PA coupling	Δ Leaflet area systole	− 0.57	0.01
ΔRV-PA coupling	Δ Tenting volume systole	− 0.73	< 0.001
ΔRV-PA coupling	Δ Tenting height systole	− 0.54	0.02
ΔRV-PA coupling	Δ Coaptation height	− 0.45	0.07
ΔRV-ESV	Annular area systole	0.67	0.002
ΔRA-Vol	Annular area systole	0.77	< 0.001

CMR-TV are delta values from morphological changes to the TV between baseline and 12 months post-operatively. r is the correlation coefficient

SPAP, Systolic pulmonary arterial pressure; CO, Cardiac output; SL, septal-lateral; Ees, End-systolic elastance; RA, Right atrium; RV, Right ventricular; PA, Pulmonary artery; ESV, End-systolic volume

Changes of TV morphology denoted by tenting volume in systole, tenting height in systole and especially tenting volume in systole correlated significantly to changes of RV-PA coupling.

## Discussion

We present, to our knowledge, the first study of TA and TV morphology assessed by CMR combined with additional invasive hemodynamic assessment in patients with severe PH due to chronic pulmonary thromboembolism during 12 months follow-up after PEA. We also provide CMR data on TA and TV morphology in normal subjects.

The main findings were as follows: Firstly, significant changes of TV morphology, noted as leaflet area, tenting volume and tenting height in systole, were significantly reduced but TV parameters were comparable to control subjects after 12 months following PEA; secondly the TA size by circumference and area were without significant changes and were comparable to control subjects.; thirdly, the TA height in systole decreased significantly and reached comparable values as compared to control subjects; fourthly, the changes to TV morphology expressed by leaflet area, tenting volume and tenting height in systole was significantly and negatively correlated to the changes in RV-PA coupling; finally, as expected significant RV and RA remodeling and improvement of RV systolic function were noted after PEA but the changes to RV and RA dimensions or volumes did not correlate to the changes of the TV morphology observed.

Preoperatively 40% of our patients demonstrated a moderate-severe functional TR in accordance with observations in PH in which severe TR varies between 10–30% of cases [2, 17]. As expected we also noted a significant reduction of moderate-severe TR with the presence of only 10% after 12 months following PEA in accordance with a previous study [8]. Tenting height is reported to be increased in TR with different etiology of PH as demonstrated by Topilsky et al. who reported a tenting height of 8.0 mm assessed by transthoracic 2D echocardiography [18]. The preoperative tenting height was significantly increased to an average of 11.4 mm by CMR (average normal subjects 7.0 mm) which normalized with a height of 7.0 mm after 12 months following PEA. In the present study, the tenting volume of the TV was increased nearly threefold preoperatively compared to controls (1.5 cm^3^) but normalized at follow-up. Increased tenting volume has been reported in patients with functional TR in two previous 3D echocardiographic studies showing a tenting volume of 4.2 cm^3^ in 17 chronic PH patients with significant TR and of 3.2 cm^3^ among 53 patients with severe TR and PH of different etiology, respectively [19, 20]. Despite different imaging techniques the tenting volume seems consistently to increase considerably in PH patients with functional TR independent of etiology. Preoperatively, we noted that the leaflet area was increased in systole to the same extent as demonstrated by Afilalo et al. in a study with patients with severe functional TR and PH. In the present study, the leaflet area was significantly decreased and normalized during follow-up^17^. A dramatic RV remodeling was shown after PEA as RV volume, and the sphericity index normalized during follow-up. Dilatation and changes of RV geometry are known to affect the position and function of the papillary muscles (PM) resulting in increased tethering of the TV [21]. Distortion of the spatial relationships between the RV, PM and TV is likely to influence the presence of TR and the demonstrated changes of the TV morphology and function. However, the major determinants of TR severity have previously been investigated by 3D echocardiography showing that leaflet area, tenting volume and leaflet closure are independent predictors of TR whereas RV dilatation or echocardiographic Doppler estimated systolic pulmonary artery pressure was not [20]. In accordance with the present findings a previous study has shown that regression of TR in PH patients is associated to the changes of TV tethering with reduction in tenting height and area as the major determinants [6].

Previous two- and three-dimensional echocardiographic studies of functional TR in PH patients have reported various degree of TA dilation depending on the severity of the regurgitation [19–21].

Among the present patients no significant increase in annular area or circumference were noted and the annulus size was comparable to control subjects. One important reason for this finding could be that the TR severity of the present population was preoperatively only moderate and thereby contributing less to RA volume overload with subsequent dilatation of the RA as well as the annulus. The potential pathophysiological mechanism in the present patient population is likely to be initiated by the remodeling of the RV and tethering of the TV as seen in other etiologies of PH [19, 22]. In the PH population with TR the TA is often less dilated as compared to patients with secondary functional TR and chronic atrial fibrillation [23, 24]. Based on the present data a conservative approach with respect to tricuspid annuloplasty in CTEPH undergoing PEA even with a significant TR seems to be advisory. In patients undergoing left valve disease surgery—in particular mitral valve repair or replacement – tricuspid annuloplasty is recommended when the TA diameter ≥ 40 mm [25].

Preoperatively, our CMR data demonstrated classic RV remodeling as seen in PH with increased.

Beyond reporting serial data on RV volume and systolic function, we were able to measure and characterize the TA and TV morphology in CTEPH patients and normal subjects by CMR. The CMR technique seems promising for TV evaluation. The benefit of CMR in the initial diagnosis of CTEPH is probably limited. However, in terms of RV characterization CMR provides very accurate estimates of RV volumes, RV function and TV regurgitant volumes. In addition, LGE and T1-mapping will probably provide insights on RV damage among CTEPH patients in the near future.

In terms of medical treatment for CTEPH there is no clear support for treatment with specific PH active drugs before PEA. Furthermore, the only approved drug in CTEPH, riociguat, is only registered for use in inoperable cases or in persistent PH after PEA [26].

Some limitations have to be considered in the present study. First of all, the number of patients studied is small but the examinations were performed prospectively and consecutively combining CMR techniques with invasively assessed hemodynamic parameters during a complete one year of follow-up. Despite the small number of patients studied as compared to other observational studies of TR and TV in PH patients, we had the opportunity to study the valve morphology in a situation where the hemodynamic conditions were reversed and thereby being able to study the associations between RV remodeling, hemodynamic and morphological TV changes.

The number of PH patients with severe TR varies considerably in line with the present findings which limits the possibility to study to what extent the observed changes in TA and TV has on the patients with severe TR.

## Conclusion

TV tenting height, volume and valve area are significantly increased whereas annulus size and shape are less effected in CTEPH patients selected for PEA when assessed by CMR. The TV morphology alterations were fully reversed and correlated to the demonstrated improvements of RV-PA coupling after PEA during 12 months of follow-up. The present data calls for further studies to evaluate the surgical strategy in CTEPH patients scheduled for PEA, as it may be possible to refrain from annuloplasty in selected cases.

## Data Availability

The imaging protocols as well as the datasets used and/or analysed during the current study are available from the corresponding author on reasonable request.

## Abbreviations

TR: Tricuspid regurgitation
TV: Tricuspid valve
TA: Tricuspid annulus
PH: Pulmonal arterial hypertension
RV: Right ventricle
CTEPH: Chronic thromboembolic pulmonary hypertension
PEA: Pulmonary thromboendarterectomy
PA: Pulmonary artery
CMR: Cardiac magnetic resonance imaging
RHC: Right heart catherization
mPAP: Mean PA pressure
TTE: Transthoracic echocardiography
ECG: Electrocardiogram
RA: Right atrium
EDV: End-diastolic volume
ESV: End-systolic volume
SV: Stroke volume
CO: Cardiac output
CI: Cardiac index
NYHA: New York Heart Association
mRAP: Mean RA pressure
PCWP: Pulmonary capillary wedge pressure
Ees: End-systolic elastance
Ea: Arterial elastance
SL: Septal-lateral
AP: Anterior–posterior
